# Feasibility of implementing an outdoor walking break in Italian middle schools

**DOI:** 10.1371/journal.pone.0202091

**Published:** 2018-08-09

**Authors:** Paolo Riccardo Brustio, Paolo Moisè, Danilo Marasso, Franco Miglio, Alberto Rainoldi, Gennaro Boccia

**Affiliations:** 1 NeuroMuscularFunction Research Group, School of Exercise and Sport Sciences, Department of Medical Sciences, University of Turin, Turin, Italy; 2 School of Exercise & Sport Sciences, SUISM, University of Turin, Torino, Italy; 3 Istituto Comprensivo Statale Buttigliera Alta-Rosta, Scuola Secondaria di primo grado “G. Jaquerio”—Buttigliera Alta, Torino, Italy; 4 CeRiSM Research Center for Sport, Mountain, and Health, University of Verona, Rovereto (TN), Italy; University of Kentucky, UNITED STATES

## Abstract

Brief bouts of physical activity during the school day are an innovative method for increasing physical activity in the school setting. The purpose of the study was to investigate if the introduction of an outdoor active break, based on walking and running, in a middle school in Italy is feasible in terms of implementation (i.e., adherence, costs, safety) and acceptability (i.e., satisfaction, intent to continue use, perceived appropriateness). One hundred and forty students (aged 12 ± 1 years) and 20 teachers (aged 50 ± 8 years) participated in the activity for four months. The activity consisted of walking (or running) one kilometer outside the school buildings during the mid-morning. Data from questionnaires regarding the satisfaction with and the acceptability of the activity were collected.

The activity was safely performed three to four days a week, without any costs to the school or to students’ parents. Students and teachers were satisfied with the activity (positive answers from 95% and 89% of each group, respectively). Moreover, the teachers reported that the program was easy to organize and did not negatively influence their teaching activities. The intervention was easily and safely implemented, and it was considered suitable for the daily routine of an Italian middle school. Further studies are needed to examine its impact on physical activity levels and academic achievement.

## Introduction

Regular participation in physical activity is considered a milestone for health and well-being at all ages, and in particular, in childhood and adolescence. Indeed, during childhood and adolescence, physical activity is important for both basic motor skills and musculoskeletal development [1,2]. Moreover, physical activity is associated with a reduction of health risks across the lifespan, such as overweight or obesity, diabetes and hypertension [3]. Despite these benefits, the amount of inactivity and sedentary behavior among children and adolescents has increased in the last years. Indeed, in Western countries (i.e., European countries and the United States) less than a third of the children follow the recommendation of performing 60 minutes of moderate-to-vigorous physical activity a day [1,2,4,5]. Interestingly, sedentary behavior increased in adolescence [6] and occurred earlier and at a higher prevalence among girls than among boys [5]. Specifically, physical activity levels decreased from ages 9 to 15 by approximately 30% and 20% in girls and boys, respectively; only half of them engaged in light physical activity, and one-quarter engaged in moderate-to-vigorous physical activity [6]. Thus, increasing daily physical activity to recommended levels is important for children’s and adolescents’ health, well-being, and healthy anthropometric parameters [7].

Regular participation in physical activity is influenced by the opportunities provided to be active. The opportunity to be active in the school systems is critical, since compulsory schooling implies that students spend a large proportion of their childhood and adolescence in the school setting [3,8]. Nevertheless, schools could be the ideal location to promote physical activity [2,3]. However, the amount of time allocated to physical activity in school settings, such as physical education, has decreased in the curriculum for different reasons in many countries [9–11]. The current increasing pressure on grade testing, the perceived lack of available time, and the perception that physical activity may threaten academic achievement seem to be the main barriers to physical activity programming in schools [12–14]. Moreover, most of the formal physical activities, apart from the physical education lessons, are supervised by classroom teachers who have no specific training in physical education [15], potentially leading to lower confidence or willingness to integrate any form of physical activity into class time. Finally, funding constraints often limit access to specialist physical education teachers and equipment.

Introducing brief physical breaks during the school day is an innovative method for increasing physical activity in school settings [16–22]. These brief bouts of physical activity, lasting generally 5–15 minutes and managed by the teacher inside the usual classroom, seem to be a feasible and efficient intervention during the school day [16–19,21,22]. For example, a 5- to 15-minute activity break seems to increase total daily step counts [16,19] and contributes to the goal of daily moderate-to-vigorous intensity physical activity [22]. Moreover, these short bouts of physical exercise seem to increase students’ attention, which is directly linked to students’ academic or cognitive performances [11]. According to the results of the meta-analysis of Erwin and colleagues [23], it is likely that physical activity interventions can be incorporated into a child’s school day to enhance learning outcomes. Importantly, a preliminary study (with 10- to 12-year-old boys) demonstrated that 10-minute breaks of physical activity conducted outside the classroom did not compromise (nor improve) participants’ on-task behavior or attention levels upon returning to the classroom [24]. This observation may encourage the inclusion of short physical activity breaks by alleviating concerns about reduced attention due to the disruption of classroom routines [24].

In particular, walking is one of the most natural forms of physical activity movement [25]. It is easy to perform and requires less skill than other activities and sports [25]. For these reasons, walking activities may be particularly suitable for children, allowing them to meet the physical activity recommendations [26,27]. Accumulated brisk walking in the primary school environment has been shown to increase energy expenditure [28] and daily physical activity levels, with a positive effect on change in body composition [29]. Using a running and walking intervention called “*The daily mile”*, Chesham and colleagues [30] found a relative increase in moderate-to-vigorous physical activities (i.e., 9 minutes per day) and a decrease in sedentary behavior (i.e., approximately 18 minutes per day) in children aged 4 to 12 years. Moreover, an increase in fitness levels, evaluated with the 20-m shuttle run test, was observed after 8 months of intervention. Another study showed that in adolescent girls, a school-based brisk walking intervention at self-selected speeds increased the amount of daily physical activity of light, but not moderate, intensity [27]. Importantly, no specific experiences in conducting physical education or sports-mediated interventions are required to participate in these kinds of programs [24]. Hence, the introduction of a brisk walking intervention in the school context may be promising, due to the inclusion of children from different socio-economic levels, which can possibly limit engagement in physical activity [30].

Summing up, brisk walking interventions during class time are promising and emerging topics due their potential for increasing the total amount of recommended physical activity in childhood and its ability to involve a large proportion of children. However, the majority of studies investigating the effects of active breaks in primary and secondary schools have been conducted outside Europe [17,24,31] or in Northern Europe [27–30]. Recently, the European Action Plan on Childhood Obesity 2014–2020 [32] underscored that Italy shows the lowest levels of physical activity for both European boys and girls in any age group. Similar results were reported in Italy from the national surveillance system *“OKkio alla Salute”*, which indicated that most children do not perform an adequate level of physical activity [33]. Hence, there is the need for more studies on this topic in Italy. Thus, the promotion of physical activity, even in the school context, should be recommended. However, despite some interventions performed in the Italian school context (e.g., [34]), no study has investigated feasibility in terms of the implementation and acceptability of a brief walking intervention during the school day in an Italian context. Accordingly, the first aim of this study was to investigate if the introduction of an outdoor active break, based on walking and running, in a middle school in Italy is feasible in terms of implementation (i.e., adherence, costs, safety) and acceptability (i.e., satisfaction, intent to continue use, perceived appropriateness). The second aim was to assess if the program positively impacts pupils’ behavior and achievement in the classroom. For these purposes, two original questionnaires, one for the students and one for the teachers, were developed and completed at the conclusion of the intervention. We think that investigating the behavior and achievement in the classroom and the feasibility of the intervention may be useful, because there are few previously published studies using this specific intervention, i.e., a walking intervention, and none of these studies were implemented in Italy.

## Materials and methods

### Participants

This intervention involved students and teachers of a middle school from the neighborhood of Turin (Buttigliera Alta, Turin) in Italy. Six classes, for a total of 142 students (F = 40%; mean age 12 ± 1 years) and 20 teachers (F = 75%; mean age 50 ± 8 years) were involved in the study. All students participated in the program, including those (n = 3) with motor, neurological, cognitive impairments. However, only students with typical development were asked to complete the questionnaires. Students’ parents were informed that participation in the study surveys was voluntary and confidential. The University of Turin institutional review board approved the study. Before the participants started the study, their parents and teachers gave their informed written consent for their participation in the study, in accordance with the ethical standards provided in the 1964 Declaration of Helsinki and Italian law.

### Activity description overview

The activity was called “*1 km a day*” and was inspired by a previous program called *“The daily mile”*, which was developed to improve opportunities for students to increase their daily physical activity in a primary school of Scotland [30,35]. The program was designed to minimize the extra work for teachers and the time impact on the curriculum. Moreover, the activity did not require any specific training for teachers since it simply constituted walking. Important features of the program were that no equipment, training or staff development were needed, and no economic contributions from either the school or the parents were required. Thus, the program was completely cost-free.

The active lesson breaks consisted of walking for one kilometer outside the school buildings along a path purposely marked in the schoolyard. The path was 350 m long, and students were required to walk it three times. The activity was supervised by the classroom teacher, who participated by walking with the students. The activity was performed in the 10 minutes just before the single break scheduled by the school at 11:00 AM.

Students and teachers participated in the activity on a daily basis for four months (from February to May 2016). During the first two weeks of the program, the number of classes involved in the program was progressively increased from one to six, in order to determine within the first days the feasibility of the organization with a small number of participants. After the completion of the program, students and teachers were requested to complete an original questionnaire. Students and teachers completed the questionnaires in the first week after the end of the intervention.

### Procedure

At the conclusion of the intervention, both students and teachers completed two different original questionnaires. The original questionnaires were designed to investigate the acceptability and feasibility of the active lesson breaks of *“1 Km a Day”* with questions about activity satisfaction and organization. Moreover, teachers collected data about the possible injuries or accidents that occurred during the activity. Both of the original questionnaires were written in Italian and translated into English for publication purposes.

### Student questionnaire

Eleven questions were developed for this study to assess the students’ perceptions about the impact of physical activity breaks on classroom behavior and on management problems due to the activity. A first part (section 1) consisted of three questions about students’ behavior, a second part (section 2) consisted of two questions about organizational aspects of the activity, and a third part consisted of four questions about satisfaction with the activity. For each item, a five-point Likert scale was used (from a minimum of 1 (strongly disagree) to a maximum of 5 (strongly agree)). Table 1 reports the eleven questions submitted to each student.

**Table 1:** Student questionnaire about perception of the activity.

#	Question	Answers
**Section 1—Student behavior**		
1	Has *“1 km a day”* increased your attention level during the second part of the morning?	1 (strongly disagree); 2;3;4;5 (strongly agree)
2	Has participation in the activity made the second part of the morning lighter?	1 (strongly disagree); 2;3;4;5 (strongly agree)
3	Has the *“1 km a day”* activity helped to facilitate relationships with your peers?	1 (strongly disagree); 2;3;4;5 (strongly agree)
**Section 2—Organizational aspects**		
4	It was difficult to follow the organizational aspects of the participation (such as changing shoes, times….)	1 (strongly disagree); 2;3;4;5 (strongly agree)
5	It was uncomfortable to interrupt the lesson for participation in the activity	1 (strongly disagree); 2;3;4;5 (strongly agree)
**Section 3 –Satisfaction**		
6	Did you consider it fun to engage in the “*1 km a day*” activity?	1 (strongly disagree); 2;3;4;5 (strongly agree)
7	Did you consider it a waste of time to engage in “*1 km a day”* activity?	1 (strongly disagree); 2;3;4;5 (strongly agree)
8	Did you wish the activity lasted more than 10 minutes?	1 (strongly disagree); 2;3;4;5 (strongly agree)
9	Did you wish the activity were proposed for the whole school year?	1 (strongly disagree); 2;3;4;5 (strongly agree)
10	Would you repeat this activity in the next school year?	1 (strongly disagree); 2;3;4;5 (strongly agree)
11	In conclusion, are you satisfied to have participated in the project?	1 (strongly disagree); 2;3;4;5 (strongly agree)

### Teacher questionnaire

Seventeen questions were developed for this study to assess the teachers’ perceptions about the impact of physical activity breaks on student behavior, organizational aspects and the activity in general. A first part (section 1) consisted of six questions about students’ behavior, a second part (section 2) consisted of four questions about the organizational aspects of the activity, and a third part consisted of five questions about satisfaction with the activity. For each item, a five-point Likert scale was used (from a minimum of 1 (strongly disagree) to a maximum of 5 (strongly agree)). Moreover, two open discretionary questions (section 4) about the positive and negative aspects of the project were included in the survey for the teachers. For more details about the online questionnaire, see Table 2.

**Table 2:** Teacher questionnaire about perception of the activity.

#	Question	Answers
**Section 1—Student behavior**		
1	Has the “*1 km a day*” activity increased the students’ attention during the second part of the morning?	1(strongly disagree); 2;3;4;5 (strongly agree)
2	Has the activity “*1 km a day*” facilitated relationships among the students?	1(strongly disagree); 2;3;4;5 (strongly agree)
3	Has the activity “*1 km a day*” facilitated relationships between you and your students?	1(strongly disagree); 2;3;4;5 (strongly agree)
4	Has it been easy to resume teaching after the break?	1(strongly disagree); 2;3;4;5 (strongly agree)
5	Has the activity negatively influenced your teaching activities?	1(strongly disagree); 2;3;4;5 (strongly agree)
6	Has the activity performance improved the academic efficiency of your students?	1(strongly disagree); 2;3;4;5 (strongly agree)
**Section 2—Organizational aspects**		
7	Were the organizational aspects easy to manage (such as change of shoes, times, etc.)?	1(strongly disagree); 2;3;4;5 (strongly agree)
8	Was it uncomfortable to interrupt the lesson to participate in the activity?	1(strongly disagree); 2;3;4;5 (strongly agree)
9	Was it easy to move the class?	1(strongly disagree); 2;3;4;5 (strongly agree)
10	Has the activity performance facilitated the second part of the morning?	1(strongly disagree); 2;3;4;5 (strongly agree)
**Section 3—Satisfaction**		
11	Did you consider it fun to perform the “*1 km a day*” activity?	1(strongly disagree); 2;3;4;5 (strongly agree)
12	Did you wish that the activity lasted more than 10 minutes?	1(strongly disagree); 2;3;4;5 (strongly agree)
13	Did you wish for there to be a proposal for the activity for the whole school year?	1(strongly disagree); 2;3;4;5 (strongly agree)
14	Would you repeat this activity in the next school year?	1(strongly disagree); 2;3;4;5 (strongly agree)
15	In conclusion, are you satisfied with participating in the project?	1(strongly disagree); 2;3;4;5 (strongly agree)
**Section 4—Open questions**		
16	What are the positive aspects of the activity “1 km a day”?	Open answer
17	What are the negative aspects of the activity “1 km a day”?	Open answer

### Data analysis

According to Carlson and collegues [36], the response options were dichotomized as agree/strongly agree (positive) vs. neutral or disagree/strongly disagree (negative). Analysis of frequencies and descriptive statistics was performed for both the students’ and the teachers’ questionnaires.

Moreover, a series of chi-square tests for each item on the questionnaires was performed in order to investigate possible gender differences in students’ responses. To describe the open questions, we decided to categorize them in relation to the different themes that emerged. Afterwards, descriptive analyses were performed in order to describe the different themes. The level of significance was set at 5% (*p <* 0.05). The Statistical Package for Social Sciences (SPSS Inc., version 24.0 for Windows, SPSS Chicago, IL, USA) was used for all statistical analyses.

## Results

### Implementation

The program was performed 3 to 4 times a week over four months. The only obstacle to the execution of the program was rainfall (36% of the possible days), during which the teachers decided not to perform the activity. No other event prevented the class from taking part in the activity. The school carried out the activity independently, without any external support. The program was completely cost-free to parents. No school resource was needed to implement the activity. No injuries or accidents occurred during the execution of the activity.

### Student questionnaire

For Section 1 of the student questionnaire, the majority of the students declared an improvement in attention (55%; M = 4 ± 1 points) and a reduction in perceived fatigue in lessons (83%; M = 4 ± 1 points) after the active break. Furthermore, the students reported that the activity favorably improved their relationships with their peers (44%; M = 3 ± 1 points). Moreover, the students did not consider the activity difficult to follow in term of management (e.g., tools and logistics) (70%; M = 2 ± 1 points) and interruption of the lesson (89%; M = 1 ± 1 points). Students considered the activity fun (89%; M = 4 ± 1 points) and not a waste of time (93%; M = 1 ± 1 points). Indeed, the 59% (M = 4 ± 1 points) of the students declared that the future program should consist of a longer activity in terms of duration (more than 10 minutes). Finally, the students showed an overall consistent satisfaction with the program (94%; M = 5 ± 1 points): the majority of students wanted to repeat the “*1 km a day*” program the next year (90%; M = 5 ± 1 points) and would implement it for the whole academic year (86%; M = 4 ± 1 points). No significant gender differences (all p-values > 0.05) were observed in relation to students’ behavior (i.e., items 1–6), organizational aspects (i.e., items 7–8) and general information about activity. For more details of the students’ responses, see Table 3.

**Table 3:** Frequencies of occurrences in relation to students’ behavior (i.e., items 1–6), organizational aspects (i.e., items 7–8) and general information about activity (i.e., items 9–11) investigated in the student questionnaire. Data are presented for the overall sample and for males and females.

Item	Answers					Overall		Males		Females		χ^2^	*p*
	1	2	3	4	5	Positive	Negative	Positive	Negative	Positive	Negative		
# 1	6%	11%	28%	35%	20%	55%	45%	53%	47%	59%	41%	0.53	0.465
# 2	4%	4%	9%	43%	40%	83%	17%	81%	19%	85%	15%	0.37	0.542
# 3	11%	20%	25%	27%	17%	44%	56%	45%	55%	42%	57%	0.06	0.807
# 4	51%	19%	16%	8%	6%	70%	30%	66%	34%	76%	24%	1.58	0.209
# 5	76%	14%	6%	4%	1%	89%	11%	86%	14%	94%	6%	2.52	0.113
# 6	0%	1%	10%	28%	61%	89%	11%	87%	13%	93%	7%	1.05	0.305
# 7	78%	15%	4%	2%	1%	93%	7%	92%	8%	94%	6%	0.36	0.551
# 8	9%	13%	19%	19%	40%	59%	41%	58%	42%	61%	39%	0.16	0.686
# 9	3%	5%	6%	22%	64%	86%	14%	87%	13%	83%	17%	0.37	0.542
# 10	2%	2%	6%	18%	72%	90%	10%	89%	11%	91%	9%	0.06	0.800
# 11	1%	1%	4%	19%	76%	94%	6%	93%	7%	96%	4%	0.69	0.408

Notes: %, percentage. For the items 4, 5 and 7, lower scores indicate “strongly agree”. Differences were calculated between males and females.

### Teacher questionnaire

For the teacher questionnaire, 80% (M = 2 ± 1 points) of the teachers reported that the program did not negatively influence their teaching activity, and furthermore, the majority of the teachers did not highlight any difficulty in resuming teaching (71%; M = 4 ± 1 points). In contrast, only 37% (M = 3 ± 1 points) of them reported that the program improved the attention and academic proficiency of the students (16%; M = 3 ± 1 points). Most teachers reported that the activity improved relationships among the students (85%; M = 4 ± 1 points) and between teachers and students (68%; M = 4 ± 1 points). The majority of the teachers reported no difficulty in moving the class for the activity (79%; M = 3 ± 1 points), while about half of them reported no problems in the logistic management of the activity (47%; M = 3 ± 1 points) and discomfort in interrupting lessons to participate in the activity (53%; M = 2 ± 1 points). Most teachers reported that the duration of the break was enough (74%; M = 2 ± 1 points). Overall, teachers were satisfied with the program (89%; M = 4 ± 1 points) and wanted to implement it for the whole academic year (65%; M = 4 ± 1 points), as well as repeat the program the next year (75%; M = 4 ± 1 points). For more details of the teachers’ responses, see Table 4.

**Table 4:** Frequencies of occurrences in relation to the observed teachers’ behavior (i.e., items 1–6), the organizational aspects (i.e., items 7–10) and the general information about the activity (i.e., items 11–15) investigated in the teacher questionnaire.

Item	Answers					Positive	Negative
	1	2	3	4	5		
# 1	10%	21%	32%	26%	11%	37%	63%
# 2	0%	10%	5%	50%	35%	85%	15%
# 3	5%	5%	21%	48%	21%	68%	32%
# 4	0%	6%	23%	53%	18%	71%	29%
# 5	60%	20%	15%	5%	0%	80%	20%
# 6	10%	32%	42%	5%	11%	16%	84%
# 7	0%	10%	42%	37%	11%	47%	53%
# 8	32%	21%	26%	16%	5%	53%	47%
# 9	0%	10%	11%	58%	21%	79%	21%
# 10	5%	28%	28%	22%	17%	39%	61%
# 11	0%	10%	5%	40%	45%	85%	15%
# 12	21%	32%	21%	11%	16%	74%	26%
# 13	0%	15%	20%	20%	45%	65%	35%
# 14	0%	10%	15%	25%	50%	75%	25%
# 15	0%	5%	5%	21%	69%	89%	11%

Notes: %, percentage; for items 5, 8 and 12, lower scores indicate “strongly agree”.

Eighteen teachers answered the open-ended question “What are the positive aspects of the activity “*1 km a day*”?” In contrast, seven teachers out of 20 did not report any responses to the question “What are the negative aspects of the activity *“1 km a day”*?*”*.

Concerning the question “What are the positive aspects of the activity *“1 km a day”*?”, the most common themes were well-being (73%; e.g., “*The “1 km a day” was useful to break the daily school routine with a healthy activity”* or *“The activity can be a method to spread the idea of the importance of carrying out daily physical activity for health”*). Moreover, another recurrent theme was about the relationship (17%; e.g., of answers: *“The activity increased the relationships among students and between students and teachers”*), the teaching (6%; e.g., “*When the students go back to the classroom*, *they are more relaxed and disciplined”*), and responsibility (6%; e.g., “*Knowing the path of the activity*, *the students were able to walk independently and responsibly*).

Concerning the question “Which are the negatives of the activity *“1 km a day”*?”, the most common themes were about time management (31%; e.g., *“Interrupting teaching activities*, *especially for teachers who have few weekly hours with the students”* or *“Subtraction of time from my teaching without concrete improvement in my subject”*) and the path (31%; e.g., *“It was little boring to repeat the same path everyday”*) aspects. Moreover, 11% of the teachers replied focusing on the organizational aspects (e.g., “*Sometimes there was a little confusion*, *especially at the beginning of the project”*), and another 11% focused on the management of the students (e.g., *“Sometimes I felt that I did not have complete control of the students along the path”*).

## Discussion

In this study, we explored the feasibility in terms of implementation (i.e., adherence, costs, safety) and acceptability (i.e., satisfaction, intent to continue use, perceived appropriateness) of a brief walking intervention in the Italian school context and its positive impacts on pupil behavior and achievement. The activity, called “*1 km a day*”, consisted of performing a physically active break in the mid-morning of walking or running for 1 km in the schoolyard. This intervention was inspired by a previous program called “*The daily mile”*, which was backed by the Scottish government [30,35]. For this purpose, two original questionnaires, one for students and one for teachers, were developed and completed at the conclusion of the intervention. However, before we discuss the results, it is necessary to point out that the present study is characterized by the evident limitations of the use of original self-report questionnaires aimed to investigate the purpose of the study. Consequently, future studies are needed in order to generalize the results for our study.

### Implementation

The high adherence to the program participation, three to four times a week, demonstrated that the activity performed during the day in middle school was successfully implemented in the context of Italian schools, as observed elsewhere [20]. The only limitation to the execution of the activity was the presence of rainfall (36% of the possible days). The activity was completely cost-free, both for parents and for the school, and it was implemented without any external support. It had the advantage of not requiring any specific training for the teachers, since it simply constituted walking. These characteristics made the program implementable without requiring the allocation of any economic resources. Moreover, no injuries occurred during the activity of the students, highlighting that the implementation of the activity was safe and without particular risks.

### Satisfaction

The perception of the activity was positive both for the students and teachers. Indeed, students, regardless of gender, and teachers would like to participate in the program during the next academic year (90% and 79%, respectively) and to extend the program throughout the whole academic year (85% and 68%, respectively). The perception of the program regarding the organization of the activity (e.g., organization, displacements and management of class time) was high both for students and teachers (ranging from 63% to 90%). Interestingly, even if a decline in physical activity levels occurred earlier and at a higher prevalence in girls than in boys [5,6], no significant difference in term of student organization and acceptability of the activity was reported. These findings underlined the successful implementation of this type of activity in the Italian school context.

Moreover, in the open-answer question, the teachers considered the daily physical activity to be important for the promotion of well-being and education. Again, the teachers considered the activity an important moment for the promotion of socialization and responsibility. Overall, the program “*1 km a day*” appeared to be a suitable intervention for increasing the amount of physical activity in middle schools in Italy.

### Pupils’ behavior

Interestingly, most of the teachers (63%) did not report a positive effect of the activity in terms of students’ attention in the second part of the morning, that is, after the execution of the active break. Conversely, previous research observed a small-to-moderate improvement in attention to tasks following physical activity breaks (effect sizes typically ranged from 0.13 to 0.60) [12]. Nevertheless, in our results, the majority of the teachers reported a neutral response to this item (see item # 1 in teacher’s questionnaire). In contrast, students, independent of gender, reported a small perceived increase in their attention level (55% positive answers to item # 1 in student’s questionnaire) after the activity, as well as a reduction in perceived fatigue in the subsequent lessons (83% positive answers; see # 2 in student’s questionnaire). Most teachers reported that the program did not impair their teaching after the break, and furthermore, that the lessons were facilitated after the break. These results are in accordance with those found in the meta-analysis of Mahar [12], which reported that physical activity performed in the classroom can improve students’ behavior and thus motivate teachers to incorporate physical activity during the school day. In contrast, a recent study demonstrated that 10-minute breaks of physical activity outside the classroom did not compromise (nor improve) participants’ attention levels upon returning to the classroom [24], showing that this activity seems to be a viable strategy for increasing physical activity without compromising academic achievement in the classroom. The above findings are important since they address the possible concerns of teachers that physical activity breaks can impair overall teaching activity, in particular after the active break. Indeed, despite the fact that a positive relation between physical activity and cognition has been widely demonstrated [37,38], this concept is difficult for teachers to accept *a priori*. However, the results of our study highlighted that teachers neither observed an improvement in the academic efficiency of their students nor a worsening (i.e., 42% neutral answers in the item # 6 of teacher questionnaire). Future studies should investigate this aspect using quantitative tests to assess attention, cognition, and academic performance to better explain these negative perceptions.

### Organizational aspects

While most teachers did not find any particular discomfort in participating in the program, few teachers (less than 10–20%) reported some criticisms regarding the interruptions and the subtraction of time from teaching activities. These concerns were particularly reported by the teachers with few lessons per week (one or two hours). Only few teachers reported that the organization of the activity was problematic because they felt that they did not have total control over the students during the activity. Thus, future programs should address the acceptability and the perceived safety of the intervention among as many teachers as possible.

### Limitations of the study

As this was an exploratory study, several limitations should be underlined. The intensity of the activity was not verified, the overall physical activity of participants was not measured, and health-related measures were not administered. Future research is needed to investigate the impact of regular participation in this kind of program on physical outcomes and on standardized cognitive and academic tests. Moreover, data were only collected from one school and did not allow us to generalize our results to a larger children’s population. Further studies should be conducted on a larger number of schools. Additionally, as previously highlighted, our study investigated the feasibility of the study with two different original questionnaires (written in the Italian language), one related to the children and one related to the teachers. We think that the implementation of new and original questionnaires could effectively contribute to better understanding of the feasibility of and satisfaction with the study. However, our results should be taken with caution. Again, the small amount of data obtained from the open questions did not allow for the application of statistical approaches (e.g., thematic analysis). Thus, future studies should measure data qualitatively (e.g., interview) to better establish acceptability and feasibility. Finally, future programs should address the acceptability and the perceived safety of the intervention among many teachers as possible.

## Conclusions

In this study, we explored the feasibility of implementing an outdoor walking break in Italian middle schools. As the children spend most of their days at school, the school is an important place for promoting strategies to increase levels of physical activity. The program, called “*1 km a day*”, consisted of performing a physically active break during the school day—walking or running 1 km outside the classroom in the schoolyard. The program was cost-free to parents and to the school. Students and teachers reported high level of satisfaction with the activity and low organizational demand. Hence, it was easily included in the school day routine among 11- to 14-year-old students.

## Supporting information

S1 Dataset Data from children enrolled in this study. (PDF)

S2 Dataset Data from teachers enrolled in this study. (PDF)
