# Supplementary material for: Feasibility of implementing an outdoor walking break in Italian middle schools
Source: PLoS One. 2018 Aug 9;13(8):e0202091. doi: 10.1371/journal.pone.0202091 (PMC6084989; doi:10.1371/journal.pone.0202091)
Supplement: S1 Dataset — (PDF) [file pone.0202091.s001.pdf]

ID_NUMBER	AGE	GENDER	#1	#2	#3	#4
1	12	M	5	5	3	1
2	12	M	3	4	4	1
3	11	F	3	4	1	2
4	11	M	5	5	5	4
5	12	M	5	5	5	3
6	11	F	4	4	4	3
7	11	M	5	5	4	3
8	11	F	5	5	5	4
9	12	M	5	5	2	4
10	11	F	2	3	5	3
11	11	M	1	2	1	2
12	11	M	4	4	2	1
13	11	M	5	5	5	5
14	11	M	3	4	4	4
15	12	F	5	5	5	1
16	11	M	3	4	3	2
17	11	F	4	5	4	2
18	12	F	4	4	3	3
19	11	M	3	3	4	2
20	11	F	4	4	4	1
21	12	F	2	3	2	1
22	11	M	1	5	4	3
23	11	M	5	4	4	1
24	12	F	4	5	4	3
25	11	M	4	4	3	3
26	11	F	3	5	2	2
27	13	M	2	1	1	5
28	13	M	3	5	3	1
29	12	F	4	5	3	1
30	12	M	3	4	3	2
31	13	M	4	1	5	1
32	13	M	4	4	3	3
33	12	M	4	5	5	1
34	12	F		4	2	2
35	13	F	2	3	1	1
36	12	F	2	4	2	1
37	12	M	3	5	4	1
38	12	M	5	5	4	5
39	13	M	1	4	5	5
40	13	M	5	5	5	1
41	12	M	4	5	2	5
42	12	F	3	5	5	1
43	12	M	2	2	1	2
44	13	M	4	5	3	5
45	12	F	5	5	5	5
46	12	M	1	3	1	4
47	12	M	2	3	5	2
48	12	M	5	4	3	3
49	13	M	4	3	3	1

50	12	F	5	5	4	2
51	12	M	3	4	2	1
52	14	M	3	5	4	1
53	13	M	4	5	4	1
54	13	F	5	5	3	1
55	13	M	4	5	4	2
56	14	F	4	4	3	1
57	14	F	4	4	4	2
58	14	M	3	4	5	1
59	13	M	3	2	2	1
60	13	F	5	5	3	1
61	14	M	5	4	3	2
62	14	M	5	4	2	5
63	14	M	3	5	4	2
64	14	F	4	4	5	1
65	14	F	3	5	2	3
66	13	F	5	5	5	1
67	13	M	3	5	1	1
68	13	M	4	5	5	2
69	14	F	4	5	4	1
70	13	M	3	1	3	1
71	14	F	1	1	4	1
72	13	M	5	4	4	4
73	13	F	2	2	2	1
74	11	M	4	5	4	1
75	11	M	2	4	4	4
76	11	F	3	5	2	3
77	12	F	4	5	3	1
78	12	F	3	4	2	4
79	11	F	3	4	2	1
80	12	M	5	4	5	3
81	12	M	3	5	3	3
82	11	F	4	4	3	1
83	11	F	1	5	1	1
84	12	M	4	4	3	1
85	12	M	4	3	4	3
86	11	F	1	5	1	1
87	11	F	4	5	5	1
88	11	M	4	4	4	1
89	11	M	3	5	5	1
90	11	F	4	5	3	3
91	12	M	4	4	4	2
92	11	M	3	4	2	1
93	11	M	3	2	3	2
94	13	F	3	4	4	1
95	11	M	3	4	2	1
96	11	F	5	3	1	1
97	11	M	3	4	3	2
98	11	M	4	4	2	1
99	11	M	4	4	2	1

100	13	F	4	4	5	2
101	13	M	3	4	4	4
102	12	F	4	5	3	1
103	12	M	2	4	1	1
104	13	F	4	4	4	3
105	12	M	2	3	1	4
106	13	M	5	5	1	1
107	12	F	4	4	2	1
108	12	M	2	1	2	1
109	12	F	4	4	3	1
110	13	M	4	5	4	2
111	12	F	2	4	2	1
112	12	M	3	5	1	1
113	12	M	4	4	2	2
114	13	M	2	4	3	3
115	12	M	4	5	2	1
116	12	M	4	4	4	1
117	12	M	4	5	4	3
118	12	M	3	4	3	1
119	13	M	4	5	4	1
120	12	M	5	5	5	1
121	13	M	3	4	1	1
122	14	M	5	5	3	1
123	13	M	5	4	3	3
124	13	M	1	3	1	1
125	14	F	3	3	3	3
126	13	F	3	4	2	1
127	14	M	4	4	3	3
128	13	M	2	5	2	1
129	14	M	4	4	4	2
130	13	F	3	2	5	1
131	13	M	3	3	5	1
132	14	F	4	4	4	1
133	13	M	4	4	3	4
134	13	F	4	4	3	2
135	14	F	5	5	2	1
136	14	F	3	4	4	3
137	13	M	3	4	3	1
138	13	F	3	4	2	2
139	13	F	4	5	4	2

#5	#6	#7	#8	#9	#10	#11	
1	5	1	5	5	5	5	
1	5	1	4	4	4	5	
1	5	1	5	5	5	5	
1	5	1	5	5	5	5	
1	5	1	4	5	5	5	
1	5	1	5	5	5	5	
1	5	1	4	5	5	5	
1	5	1	3	3	5	5	
1	4	1	3	5	5	5	
1	5	1	5	5	5	5	
1	5	2	5	5	4	5	
1	4	2	2	3	4	4	
1		1	3	5	5	5	
1	5	1	3	5	5	5	
1	5	1	4	5	5	5	
1	5	1	3	4	5	5	
2	5	1	4	5	5	5	
1	4	1	5	5	5	5	
1	5	1	4	3	5	5	
1	4	2	5	5		4	
2	3	3	1	2	4	4	
4	5	1	1	5	5	4	
1	5	1	5	5	5	5	
1	5	1	5	5	5	5	
1	5	1	5	5	5	5	
1	5	1	2	5	5	5	
4	3	4	1	1	1	1	
1	5	1	5	5	5	5	
1	4	1	2	5	5	4	
2	3	1	4	1	5	5	
1	5	1	1	5	5	5	
1	5	1	4	5	5	5	
1	5	1	5	5	5	5	
2	5	1	3		5	4	
1	3	3	2	3	3	3	
1	5	1	5	5	5	5	
1	5	1	5	5	5	5	
5	4	1	5	5	5	5	
1	5	3	2	1	3	5	
1	5	1	3	5	5	5	
1	5	2	3	2	4	5	
1	5	1	4	5	5	5	
1	5	1	5	3	4	3	
1	5	4	3	4	3	4	
1	5	1	5		5	5	
4	2	4	1	2	2	2	
1	4	1	3	4	5	5	
1	5	1	3	4	5	5	
3	4	1	2	4	4	5	

1	5	1	5	5	5	5
1	3	2	1	4	4	3
1	5	1	1	5	5	5
1	5	1	5	5	5	5
3	4	1	2	4	5	5
1	5	1	5	5	5	5
1	4	1	2	3	4	4
1	4	1	5	4	5	5
1	5	1	5	5	5	5
1	3	1	2	4	4	4
1	4	1	3	5	5	5
3	3	3	5	4	3	3
2	4	1	3	4	4	5
3	5	1	5	5	5	4
2	5	2	3	5	5	5
1	4	1	4	5	5	4
1	5	1	5	5	5	5
1	5	1	1	5	5	5
2	5	1	4	5	5	5
1	5	1	5	5	5	5
1	5	1	5	5	5	4
1	3	2	3	2	4	3
1	4	5	4	5	5	5
1	4	1	5	4	5	5
4	5	1	5	5	5	5
3	5	1	2	4	5	4
1	4	1	5	5	5	5
1	5	1	2	5	5	5
1	4	2	4	5	5	5
1	4	3	1	4	4	5
1	4	1	5	5	5	4
1	4	1	5	5	5	5
1	5	1	5	5	5	5
1	5	2		5	5	5
1	5	1	2	5	5	5
2	5	2	1	2	5	5
1	5	2	5	5	5	5
1	5	1	4	4	5	5
1	5	2	4	4	5	5
1	5	1	5	5	5	5
2	4	1	4	5	5	4
1	5	2	5	5	5	5
1	4	1	2	4	5	5
3	3	1	4	4	3	4
1	4	1	4	5	5	5
1	5	1	5	5	5	5
1	4	1	5	3	2	4
1	5	1	3	5	5	5
1	5	1	3	5	5	5
2	5	1	2	4	4	5

1	5	1	5	5	5	5
1	5	1	5	5	4	5
1	5	1	5	5	5	5
1	4	2	4	4	2	3
4	5	1	3	5	3	4
1	4	1	4	5	5	5
1	4	1	4	5	5	4
1	5	1	3	4	4	5
1	3	3	5	5	5	5
1	4	1	3	5	5	5
2	4	2	3	4	4	4
1	3	1	2	1	1	5
1	5	1	5	5	5	5
2	4	1	4	4	4	5
1	5	1	3	1	4	5
2	3	2	2	4	5	4
1	5	1	5	4	5	5
3	5	2	5	5	5	5
1	5	1	5	5	5	5
2	4	1	5	5	5	5
1	5	1	4	5	5	5
1	3	1	2	5	3	4
1	4	1	3	5	4	5
2		1	5	4	5	5
2	3	2	3	4	4	4
2	4	1	3	3	3	4
1	5	1	3	4	4	5
3	4	2	1	2	1	5
1	4	2	4	5	4	5
2	5	1	3	5	5	5
1	4	1	2	5	5	5
1	5	1	5	5	5	5
1	5	1	5	5	5	5
1	5	1	5	5	5	4
2	4	1	4	5	5	5
1	5	1	5	5	5	5
1	5	1	1	2	4	5
1	5	1	5	5	5	5
1	4	2	4	4	4	4
3	5	1	5	5	5	5
