# Supplementary material for: Feasibility of implementing an outdoor walking break in Italian middle schools
Source: PLoS One. 2018 Aug 9;13(8):e0202091. doi: 10.1371/journal.pone.0202091 (PMC6084989; doi:10.1371/journal.pone.0202091)
Supplement: S2 Dataset — (PDF) [file pone.0202091.s002.pdf]

ID_NUMBER	Age	Gender	#1	#2	#3	#4	#5	#6	#7	#8	#9
Teacher 1	41	F	1	4	5	4	1	1	3	4	5
Teacher 2	41	F	2	4	3	3	1	2	2	3	2
Teacher 3	57	F	3	4	3	4	1	2	4	1	4
Teacher 4	41	F	4	5	4	5	1	3	3	1	4
Teacher 5	54	F	3	4			3	3	3	4	2
Teacher 6	44	M	2	4	4	4	1	3	3	1	4
Teacher 7	62	M	4	5	5	4	1	5	3	2	4
Teacher 8	46	M	2	4	4	3	2	2	3	2	3
Teacher 9	62	F	1	2	1	2	4	1	3	5	4
Teacher 10	55	F	3	4	3	3	1	3	5	1	5
Teacher 11	62	F		4	4		2	2	4	3	4
Teacher 12	50	F	3	5	4	4	1	3	5	1	5
Teacher 13	55	F	3	5	5	3	3	3	4	3	4
Teacher 14	47	F	5	5	4	5	1	4	4	1	4
Teacher 15	42	F	4	4	3	4	2	3	4	3	3
Teacher 16	52	M	5	5	5	5	1	5	4	2	5
Teacher 17	56	F	4	5	4	4	2	3	3	2	4
Teacher 18	60	F	3	3	4	4	1	2	4	4	4
Teacher 19	41	M	2	2	2	4	3	2	2	3	4
Teacher 20	41	F	4	4	4		1				

#10	#11	#12	#13	#14	#15	#16	#17
1	5	1	3	4	5	health	path
2	4	3	3	3	3	relationship	logistic
3	4	4	5	5	5	relationship	path
4	5	5	5	5	5	health	path
3	2	2	3	3	4	health	path
2	5	2	5	5	5	health	Menagment of student
4	5	5	5	5	5	health	Menagment of student
2	4	3	4	5	5	promotion	study
2	2	1	2	2	2	health	study
5	4	2	5	5	5	health	
	4	2	2	3	4	health	
3	5	2	4	4	5	responsability	logistic
3	5	3	5	5	5	relationship	
5	5	4	5	5	5	health	
4	4	1	3	4	4	health	study
5	5	5	5	5	5	school	
4	5	3	5	5	5	health	path
3	4	1	4	4	4	health	study
2	3	2	2	2	5		
	4		4	4			
